# Aqueous olefin metathesis: recent developments and applications

**DOI:** 10.3762/bjoc.15.39

**Published:** 2019-02-14

**Authors:** Valerio Sabatino, Thomas R Ward

**Affiliations:** 1 Department of Chemistry, University of Basel, Building 1096, Mattenstraße 24a, Biopark Rosental, 4058, Basel, Switzerland

**Keywords:** aqueous catalysis, artificial metalloenzymes, chemical biology, green chemistry, olefin metathesis, ruthenium catalysts, stapled peptides

## Abstract

Olefin metathesis is one of the most powerful C–C double-bond-forming reactions. Metathesis reactions have had a tremendous impact in organic synthesis, enabling a variety of applications in polymer chemistry, drug discovery and chemical biology. Although challenging, the possibility to perform aqueous metatheses has become an attractive alternative, not only because water is a more sustainable medium, but also to exploit biocompatible conditions. This review focuses on the progress made in aqueous olefin metatheses and their applications in chemical biology.

## Introduction

**Scheme 1:**
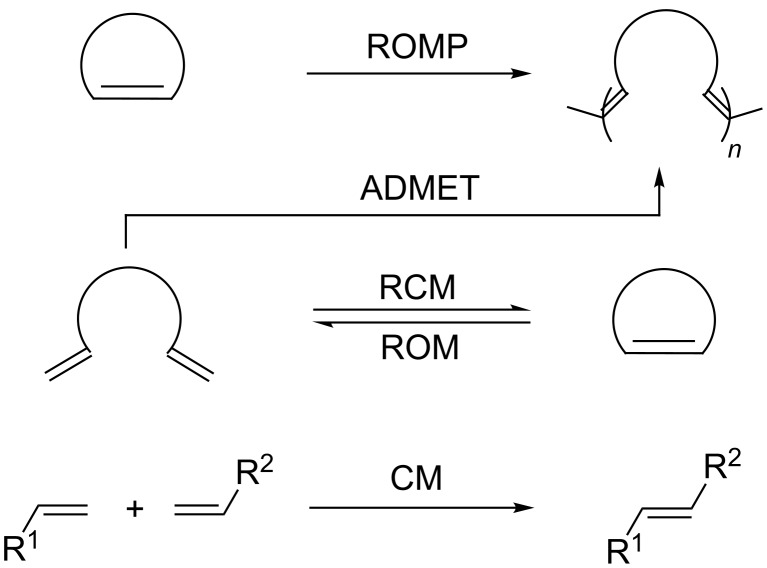
Most common metathesis reactions. Ring-opening metathesis polymerization (ROMP), acyclic diene metathesis (ADMET), ring-closing metathesis (RCM), ring-opening metathesis (ROM), and cross-metathesis (CM).

Olefin metathesis represents a versatile synthetic tool for the construction of carbon–carbon bonds [1–9]. Since its first report in 1956, a Ti(II)-catalyzed polymerization of norbornene [10], metathesis rapidly attracted interest among organic chemists and has been used in different research fields spanning polymer chemistry [11–12] to drug discovery [13–15]. Scheme 1 displays the most common metathesis reactions.

**Scheme 2:**
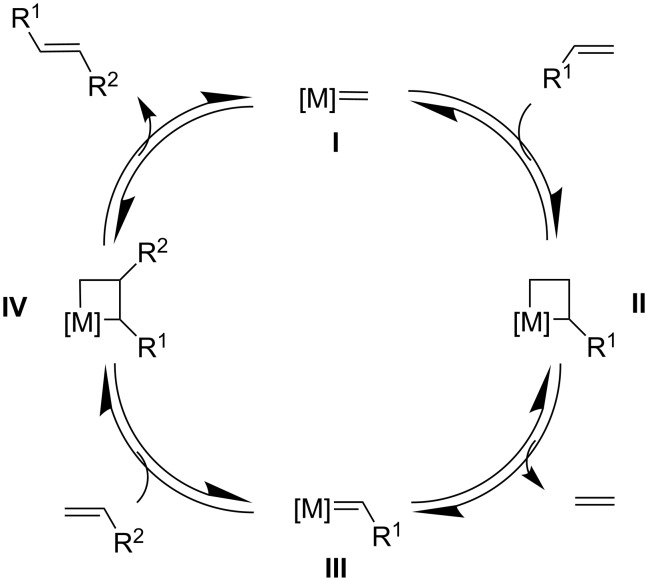
Catalytic cycle for metathesis proposed by Chauvin.

The metathesis reaction mechanism, proposed by Chauvin in 1971, suggests that the reaction proceeds via the reversible formation of a metallacyclobutane intermediate (Scheme 2, intermediates **II** and **IV**) [16]. The catalytic cycle involves an initial [2 + 2] cycloaddition between a metal carbene **I** and an olefin, followed by a retro [2 + 2] cycloaddition, leading to the release of a “scrambled” olefin (e.g., ethylene in Scheme 2) and the metal carbene species **III** as key intermediate. A [2 + 2] cycloaddition with a second olefin leads to the formation of intermediate **IV**, followed by a retro [2 + 2] cycloaddition that regenerates catalyst **I** and releases the metathesis product. This visionary mechanistic proposal was later confirmed by experimental studies [17–20].

**Figure 1:**
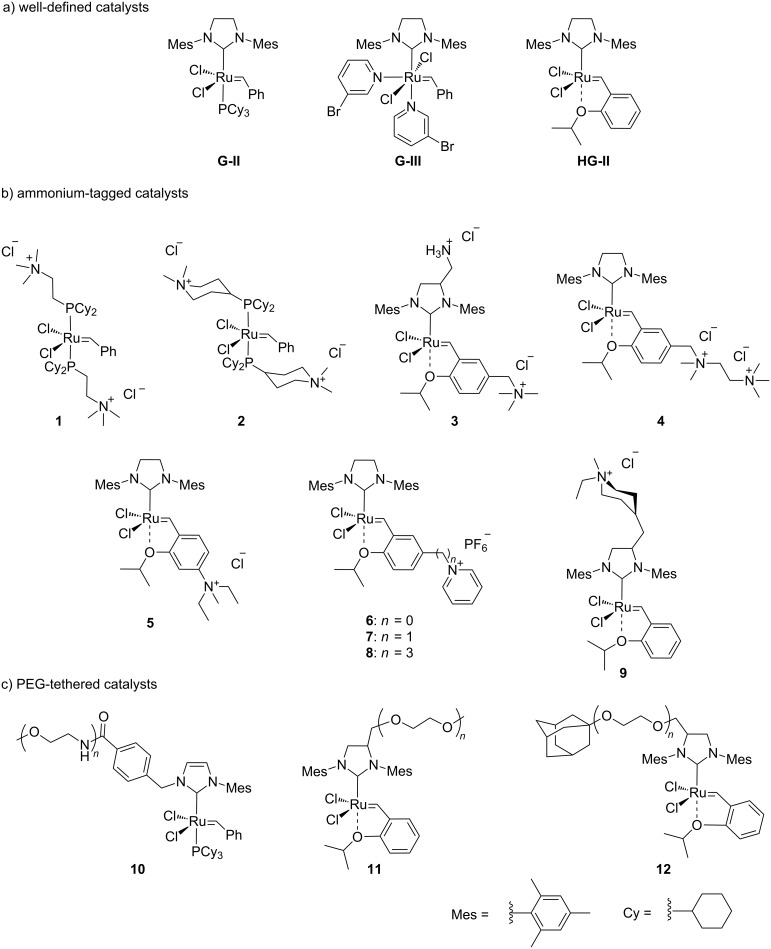
Some of the most representative catalysts for aqueous metathesis. a) Well-defined ruthenium catalysts. b) Catalysts bearing ammonium tags. c) PEG-tethered catalysts.

Ruthenium-based catalysts are among the most tolerant and stable metathesis catalysts and are widely employed for metatheses in aqueous media [21–22]. There is a growing interest in performing metathesis reactions in water as a greener alternative to chlorinated or aromatic solvents [23–24]. Water is inexpensive, non-flammable, non-toxic and environmentally friendly, all characteristics that make it an ideal solvent. Furthermore, water is the media of biochemical reactions, and metathesis is a bioorthogonal reaction that can be exploited in a biological setting. Figure 1 illustrates some of the most representative catalysts developed for aqueous metathesis. Water-soluble catalysts are obtained by derivatization of classical catalysts **G-II** and **HG-II** (Figure 1a), resulting from the introduction of ionic tags and highly polar groups such as ammonium tags (Figure 1b) and PEGs (Figure 1c). This review focuses on the recent improvements of olefin metathesis in aqueous media and the resulting applications in bioinorganic chemistry and chemical biology.

## Review

### Challenges in aqueous metathesis

**Scheme 3:**
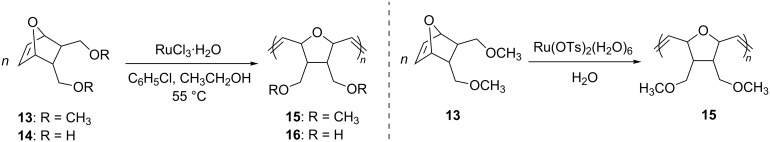
First aqueous ROMP reactions catalyzed by ruthenium(III) salts.

The first examples of aqueous metathesis were reported in the late 1980s [25–26]. ROMP reactions of 7-oxanorbornene derivatives **13** and **14** were carried out with the so-called “ill-defined” catalysts, namely RuCl_3_·H_2_O and Ru(OTs)_2_(H_2_O)_6_ [27–28] (Scheme 3). However, these catalysts had limited usefulness due to a slow initiation rate and detrimental effect of water on the reaction mixture.

**Scheme 4:**
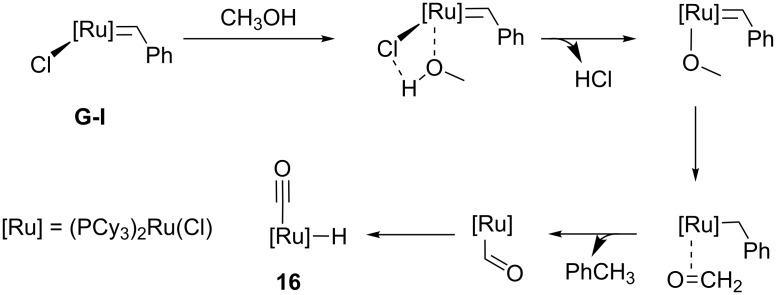
Degradation pathway of first generation Grubbs catalyst (**G-I)** in methanol.

Water can lead to the formation of catalytically inactive Ru hydride species. Fürstner et al. isolated these complexes as byproducts during the synthesis of Grubbs second generation-type catalysts with saturated NHC ligands [29]. In this specific case, the formation of the metal hydride complex is believed to occur during the work-up with methanol. Dinger and Mol also carried out studies supporting this theory [30]. In their report, they elucidated the degradation pathway of the first generation Grubbs catalyst (**G-I**) in the presence of primary alcohols and water (Scheme 4). The detrimental effect of water is more likely to occur at high temperatures and in the presence of a base.

^1^H NMR studies revealed that methanol is the source of hydride and this was later confirmed by Grubbs and co-workers [31]. The proposed mechanism for the degradation of **G-I** occurs via alcohol dehydrogenation followed by decarbonylation of the ruthenium hydride **16**.

In 2015, Cazin and co-workers showed that the detrimental effect of H_2_O also occurs with the more innovative catalysts **Caz-I**, **Ind-II** and **HG-II** (Table 1) [32]. The authors performed the RCM of the challenging substrate **17** in toluene at 110 °C, reporting excellent yields in reactions carried out on a benchtop under air using non-degassed technical-grade solvents. However, upon addition of 100 µL of distilled degassed water to the reaction mixture, the conversions dropped to 36%, 15% and 8%, respectively, for **HG-II**, **Caz-I**, and **Ind-II** (Table 1). Thus, the presence of H_2_O (ca. 6%) severely affects the phosphine-based catalysts **Caz-I** and **Ind-II**, while it has a less pronounced detrimental effect on the isopropyloxy-benzylidene catalyst **HG-II**.

**Table 1:** RCM of challenging substrate 17 in air and in the presence of water.

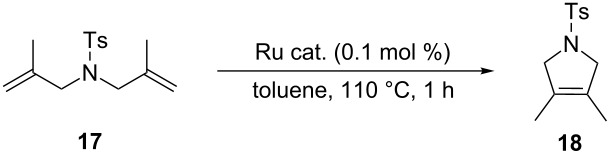				

Ru cat.		dry air (conv. %)	air (conv. %)	H_2_O (conv. %)

**Caz-I**	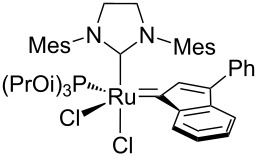	90	60	15

**Ind-II**	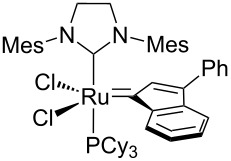	70	22	8

**HG-II**	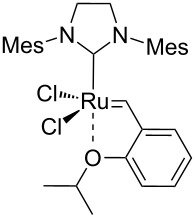	60	38	36

### “On water” vs “in water” metathesis

**Scheme 5:**
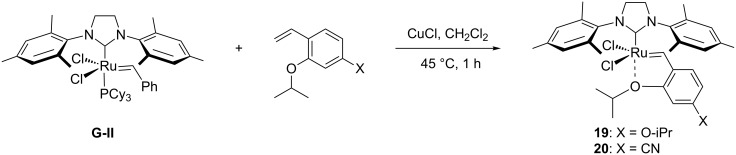
Synthesis of Blechert-type catalysts **19** and **20**.

Hydrophobic catalysts are able to perform metathesis in aqueous mixtures. Blechert and Raines reported examples of RCM, CM and ROMP in heterogeneous conditions with hydrophobic catalysts [21,33]. Blechert prepared alkoxy- and cyano-substituted catalysts **19** and **20** from **G-II** (Scheme 5) [34], while Raines and co-workers employed the conventional catalysts **G-II** and **HG-II** [35].

Blechert and Raines both performed RCM reactions with the benchmark substrate **21** in mixtures of water/organic solvent at room temperature in air (Table 2).

**Table 2:** RCM of *N,N*-diallyltoluenesulfonamide (**21**) with ruthenium catalysts.

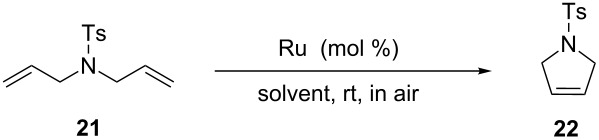					

catalyst (mol %)	solvent	*t* (h)	*T* (°C)	conv. (%)	reference

**G-II** (5) **G-II** (5) **G-II** (3) **G-II** (3) **G-II** (3) **9** (3) **9** (3) **9** (3) **HG-II** (3) **HG-II** (1)	acetone/H_2_O 2:1 THF/H_2_O 4:1 MeOH/H_2_O 3:1 MeOH/H_2_O 1:3 DMF/H_2_O 1:3 MeOH/H_2_O 3:1 MeOH/H_2_O 1:3 DMF/H_2_O 1:3 acetone/H_2_O 2:1 DME/H_2_O 2:1	24 24 12 12 12 12 12 12 2 24	rt rt 22 22 22 22 22 22 rt rt	>95 3 29 77 82 87 94 94 >95 95	[35] [35] [34] [34] [34] [34] [34] [34] [35] [35]

Table 2 summarizes the activities of the different ruthenium catalysts in protic media. The ratio water/co-solvent affects the RCM of substrate **21** catalyzed by **G-II** (77% conversion in MeOH/H_2_O 1:3 and 29% conversion in MeOH/H_2_O 3:1). The drastic loss of activity can be traced back to the better activity of **G-II** under aqueous-emulsion conditions and the poor solubility of **G-II** in MeOH. These results suggest how important the role of the hydrophobic effect is on the catalytic activity of the reaction. In fact, catalyst and substrate are encapsulated into emulsion droplets formed in the reaction media above the aqueous layer, making the reaction proceed “on water” [21–22].

**Figure 2:**
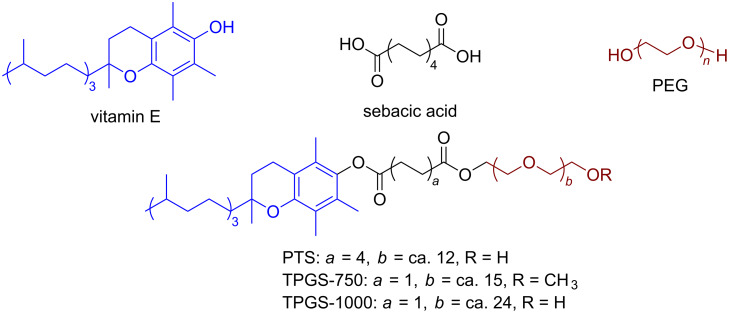
Chemical structure and components of amphiphilic molecule PTS and derivatives.

The introduction of amphiphilic molecules for aqueous micellar catalysis allows metathesis to proceed efficiently “in water” [36]. Lipshutz and co-workers generalized the application of a three-component non-ionic surfactant for numerous reactions in water, including olefin metathesis [37–39]. The surfactant, PTS, incorporates α-tocopherol, sebacic acid and PEG moieties as part of its structure, resulting in a non-ionic amphiphile (Figure 2).

**Scheme 6:**
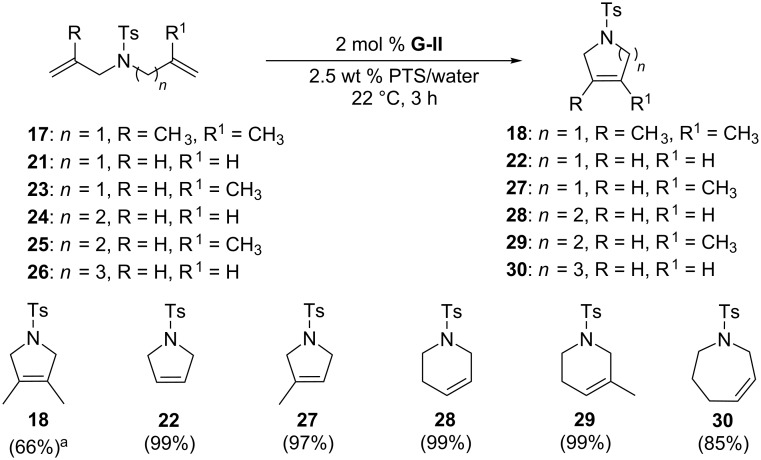
RCM of selected substrates in the presence of the surfactant PTS. Conditions^a^: The reaction was carried out at 60 °C for 24 hours.

In water, PTS forms nanomicelles which contribute to the solubilization of water-insoluble substrates and catalysts, thus contributing significantly to improve olefin metathesis yields. The positive effect of this strategy was demonstrated by Lipshutz and co-workers for RCM and for CM reactions [40–41]. Scheme 6 displays the RCM of selected substrates with **G-II** as catalyst in the presence of PTS as surfactant. The work of Lipshutz and co-workers is extensively reported elsewhere [21,33,42–43].

**Scheme 7:**
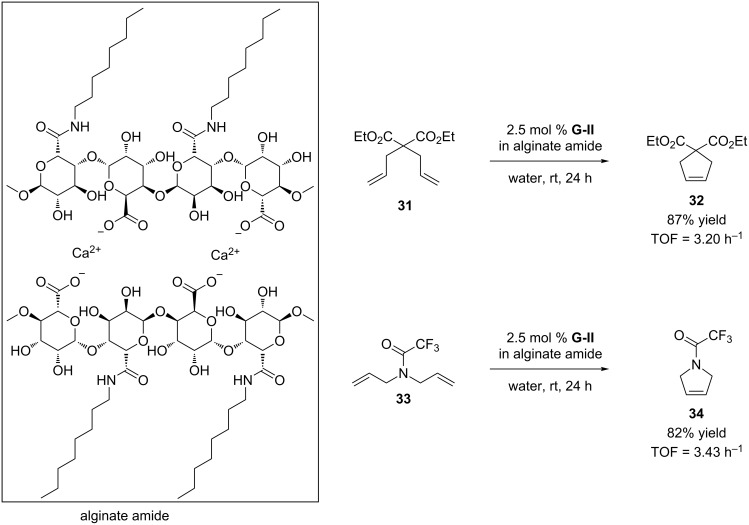
RCM reactions of substrates **31** and **33** with the encapsulated **G-II** catalyst.

Catalyst encapsulation is a recent example of “in water” metathesis with a heterogenous catalytic system. Pauly et al. used alginate beads as a matrix to encapsulate the **G-II** catalyst for the RCM of substrate **31** and **33** (Scheme 7) [44]. Alginate amide beads perform best in neat water as they facilitate the diffusion of hydrophobic substrates through the beads. However, the reaction rates are very low compared to the non-encapsulated catalyst **G-II**. The main advantage of the catalyst encapsulation is the catalyst recycling, as the alginate beads can be reused up to 10 times, retaining about 80% of activity.

### Catalysts bearing quaternary ammonium tags

Classical metathesis catalysts such as **G-II** and **HG-II** are among the most active, stable and versatile ruthenium complexes. Despite their high activity and remarkable stability, they are sparingly soluble in neat water, thus challenging their use as homogeneous catalysts in pure water. To overcome this challenge, a small amount of organic co-solvent (or surfactant) is frequently used.

The removal of residual ruthenium traces is a crucial step for most industrial applications [45–50]. Indeed, the purification of products from metathesis reaction mixtures often requires multiple tedious steps, primarily because metal complexes’ impurities in the final product may cause isomerization or decomposition of the products and may be toxic. The latter is a very critical issue for the pharmaceutical industry, as the amount of ruthenium in APIs (active pharmaceutical ingredients) may not exceed 100 µg/day for drugs administered per os (oral administration) and 1 µg/day by inhalation [51].

Some of the difficulties highlighted above can be overcome by the incorporation of quaternary ammonium tags, which simplify product purification as well as olefin metathesis in pure water [52–53].

**Scheme 8:**
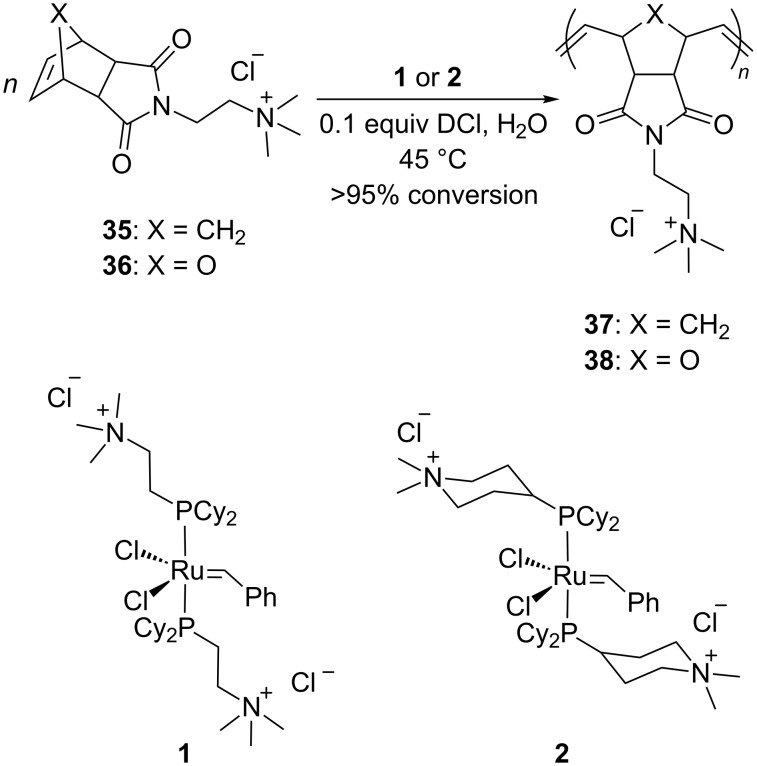
Living ROMP of norbornene derivatives **35** and **36** with phosphine-based catalysts bearing quaternary ammonium tags **1** and **2**.

Grubbs and co-workers were the first to introduce water-soluble catalysts which displayed metathesis activity in aqueous media [54]. In 1996, Grubbs et al. reported that complexes **1** and **2** catalyze the living opening polymerization of norbornene derivatives **35** and **36** in neat water. Interestingly, the presence of a Brønsted acid led to the protonation of one phosphine ligand rather than reacting with the ruthenium alkylidene moiety. Scavenging of the trialkylphosphine moiety resulted in a more active complex capable of initiating the ROMP of 2,3-difunctionalized norbornadienes and 7-oxo analogues (Scheme 8).

**Scheme 9:**
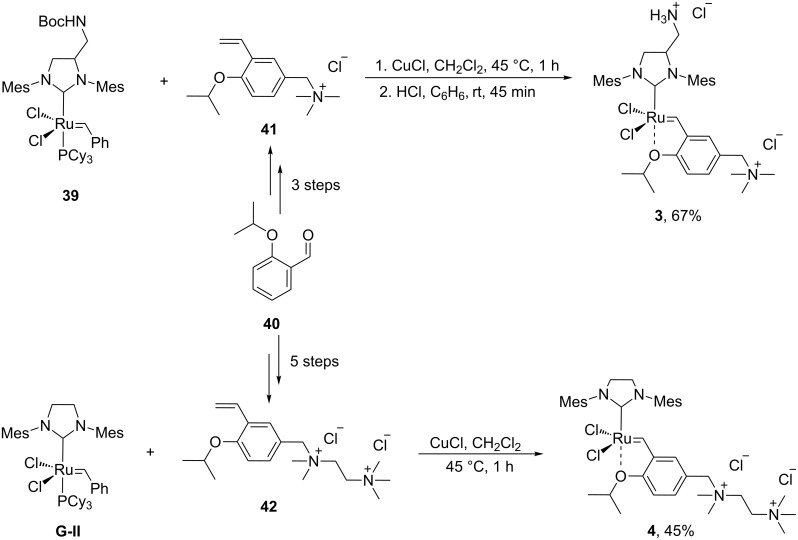
Synthesis of water-soluble catalysts **3** and **4** bearing quaternary ammonium tags.

However, catalysts **1** and **2** are unstable in water and their use is limited to ROMP. Polyethylene glycol (PEG)-tagged catalysts (**10** and **11**, Figure 1c) showed significantly improved RCM activities in water, but they tend to form aggregates in water due to their high molecular weight (ca. 5,000 g·mol^−1^) [55]. A few years later, Grubbs and co-workers reported the use of NHC complexes containing quaternary ammonium tags [56]. The catalysts **3** and **4** were obtained by the reactions of **G-II** and the asymmetric Boc-protected derivative **39** with 2-isopropyloxystyrene derivatives **41** and **42** (Scheme 9). Catalysts **3** and **4** showed modest activities in the ROMP of substrate **35**.

**Scheme 10:**
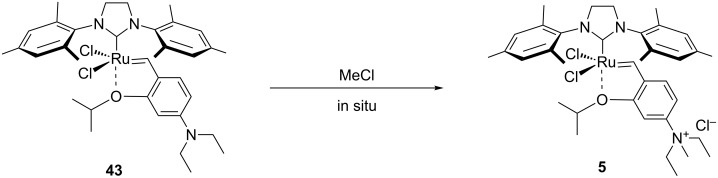
In situ formation of catalyst **5** bearing a quaternary ammonium group.

In 2006, Grela and co-workers reported the synthesis of the metathesis catalyst **5** also bearing a quaternary ammonium tag [57]. Following their previous studies highlighting the beneficial effect of an electron-withdrawing group (EWG) on the benzylidene moiety, such as NO_2_ [58], they proposed an “electron-donating to electron-withdrawing activity switch”, consisting of an in situ formation of quaternary ammonium salts by treatment with Brønsted acids (Scheme 10). Several metathesis reactions were performed in methanol/water mixtures with EWG-substituted catalyst **5**.

The “in situ” strategy was successfully applied to the preparation of catalysts **47**, **48** and **49** by Skowerski et al. [59]. Treatment of the free bases **44**, **45** and **46** with methyl chloride (MeCl) yielded the corresponding ammonium quaternized groups (Table 3).

**Table 3:** The “in situ” formation of quaternary ammonium-tagged catalysts.

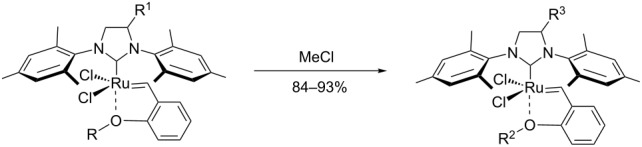					

precursor	R	R**^1^**	catalyst	R**^2^**	R**^3^**

**44**	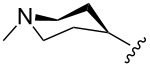	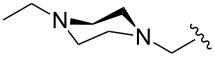	**47**	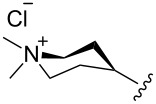	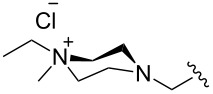

**45**	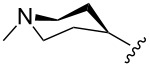	H	**48**	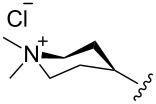	H

**46**	iPr	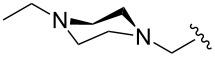	**49**	iPr	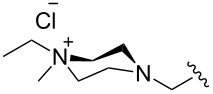

In a recent publication, catalyst **9** was used for an aqueous living ring-opening metathesis polymerization-induced self-assembly (ROMPISA). The authors demonstrated the possibility of performing living ROMP in water selecting a quaternary ammonium-based phenyl norbornene carboximide as core-forming monomer [60]. This polymer is currently being investigated for possible biomedical applications.

Table 4 summarizes the activities of the different ammonium-tagged catalysts discussed above with several water-soluble substrates. Catalysts **3** and **4** showed modest to excellent activities in the RCM of *N*,*N*-diallylated substrate **50** (respectively 36% and >95% yield with **3** and **4**) and substrate **54** (>95% yield with both catalysts). There is no obvious explanation why the RCM of **52** does not occur under identical conditions. Catalysts **9**, **47** and **48** display good activities for the ring-closing of substrates **54** and **56**, for the self-metathesis of allyl alcohol (**59**) and the *cis*–*trans* isomerization of *cis*-butenedienol (*Z*-**58**).

**Table 4:** Aqueous metathesis of selected substrates with water-soluble catalysts bearing quaternary ammonium groups.

substrate	product	cat. (mol %)	*T* (°C)	*t* (h)	yield % (*E*:*Z*)

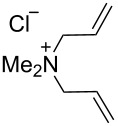 **50**	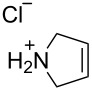 **51**	**3** (5) **4** (5)	rt rt	4 24	36 >95
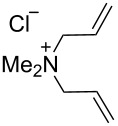 **52**	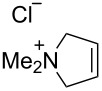 **53**	**3** (5) **4** (5)	rt rt	24 24	<5 <5
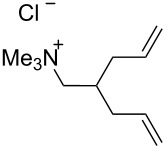 **54**	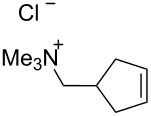 **55**	**3** (5) **4** (5) **9** (5) **47** (5) **48** (5)	rt rt rt rt rt	12 24 2.5 2.5 3.5	>95 >95 96 88 49
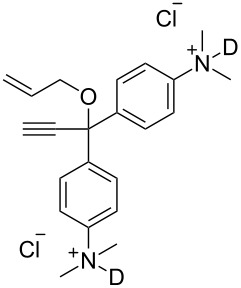 **56**	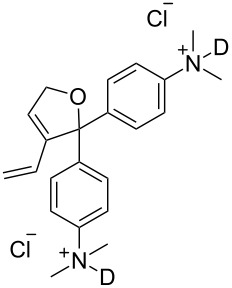 **57**	**9** (5) **47** (5) **48** (5)	rt rt rt	5 5 5	46 41 62
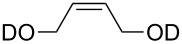 ***Z*-58**	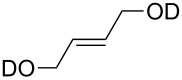 ***E*-58**	**3** (5) **4** (5) **9** (0.5) **47** (0.5) **48** (0.5)	30 30 rt rt rt	2 24 0.16 0.13 1.1	94 92 94 94 71
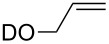 **59**	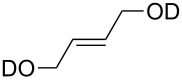 ***E/Z*** **58**	**3** (5) **4** (5) **9** (5) **47** (5) **48** (5)	45 45 rt rt rt	6 24 24 24 24	69 82 77 (16.7:1) 38 (12.5:1) 74 (16.7:1)

**Scheme 11:**
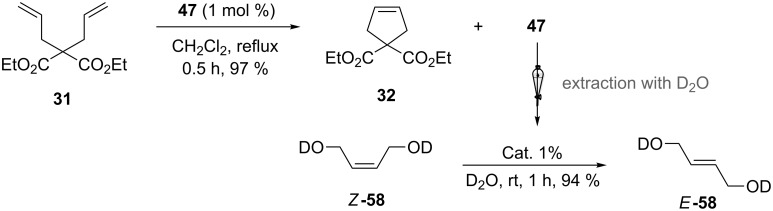
Catalyst recycling of an ammonium-bearing catalyst.

Metathesis catalysts bearing quaternary ammonium groups provide an attractive alternative to classical ruthenium catalysts. Although they do not represent a great improvement in terms of catalytic activity, they significantly improve the water solubility and facilitate the removal of ruthenium residues from reaction mixtures [52,59]. The majority of such ruthenium complexes can easily be removed, especially for the metathesis of water-insoluble substrates, as demonstrated by Grela and co-workers for the RCM of diallylmalonate **31** in DCM (Scheme 11). Upon reaction completion, the catalyst is extracted from the organic reaction mixture with D_2_O and (re)-used for the isomerization of *cis*-butenediol *Z*-**58** in water.

**Scheme 12:**
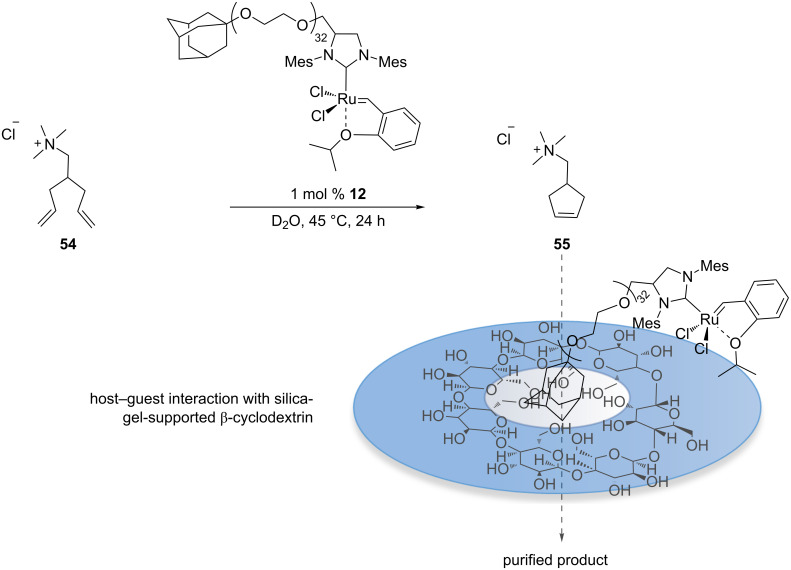
Removal of the water-soluble catalyst **12** through host–guest interaction with silica-gel-supported β-cyclodextrin.

Recently the removal of a water-soluble catalyst from reaction mixtures was also achieved with catalyst **12** (Figure 1c) through host–guest interactions [61]. Chung and co-workers used a PEG-tethered adamantyl ligand for various metathesis reactions in water and DCM [62]. The authors showed that the catalyst can be easily removed by generating a host–guest complex between silica-grafted β-cyclodextrin and the adamantyl group of catalyst **12**. A simple filtration of the crude mixture through a cotton plug after RCM of substrate **54** yields the purified product with 53 ppm of residual ruthenium (Scheme 12).

### Metathesis with artificial metalloenzymes

**Scheme 13:**
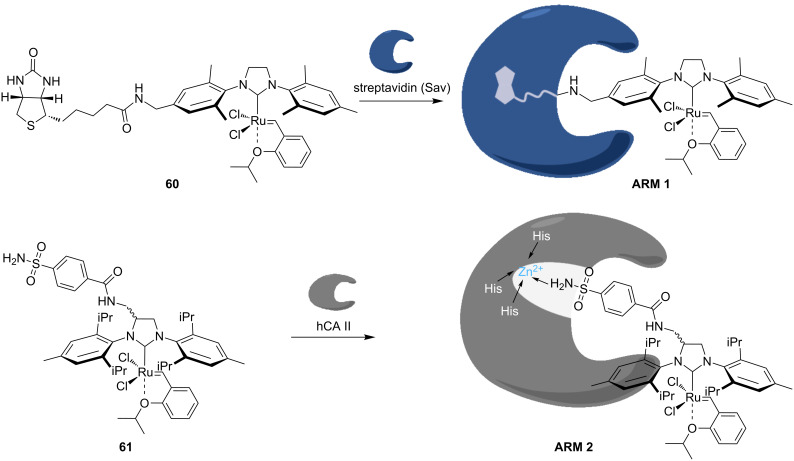
Selection of artificial metathases reported by Ward and co-workers (**ArM 1** based on biotin–(strept)avidin technology and **ArM 2** based on dative anchoring to hCAII).

Directed evolution allows an iterative improvement by successive rounds of mutation and screening the performances of genetically-encoded enzymes. Hypothesizing that this tool may be applicable to the optimization of artificial metalloenzymes (ArMs) for olefin metathesis, a new-to-nature bioorthogonal reaction might be introduced in a biological system. ArMs result from the incorporation of a catalytically active organometallic moiety within a protein scaffold. Such biohybrid catalysts enable a chemogenetic optimization of their catalytic performances. As olefin metathesis is bioorthogonal, it offers attractive features for the manipulation of biological systems. Comprehensive reviews on ArMs can be found elsewhere [63–64]. Several artificial metalloenzymes able to perform metathesis, coined artificial metathases, have been reported since 2011. The artificial metathases rely on different strategies to anchor the organometallic moiety to the protein scaffold and include supramolecular, dative, as well as covalent anchoring. Ward and co-workers reported the first artificial metathase based on the biotin–(strept)avidin technology in 2011 [65], thus expanding the set of reported reactions with this class of ArMs [66]. It is well known that the biotin–(strept)avidin couple possesses one of the highest non-covalent binding affinities (*K*_d_ = 10^−12^–10^−15^ M). This exceptional affinity warrants the ArM remaining assembled throughout catalysis. Biotinylated HG-type catalysts anchored within (strept)avidin through supramolecular interactions were tested in the RCM of *N*,*N*-diallyltoluenesulfonamide (**21**) in aqueous media, achieving encouraging results at pH 4 and in the presence of MgCl_2_ [65]. The chemical optimization of the organometallic moiety revealed catalyst **60**, which was combined with streptavidin (Sav) to afford **ArM 1** (Scheme 13). Ward and co-workers reported another artificial metathase based on the dative anchoring of a biotinylated HG-type catalyst to human carbonic anhydrase II (hCAII) in 2015 [67]. The active site of hCAII contains Zn^2+^ which is coordinated to three histidines. Catalyst **61** contains an arylsulfonamide moiety that coordinates the metal with high affinity (*K*_d_ = 205 nM), affording **ArM 2** (Scheme 13).

From the different organometallic moieties tested, the catalyst containing 2,6-diisopropylphenyl groups on the NHC ligand afforded the highest activity for the aqueous RCM of *N*,*N*-diallyltosylamine (**21**). Metathase **ArM 2** performed best in phosphate buffer at pH 5.0, yielding 85% of product **22** (Table 5). A substitution of lysine with histidine at position 198 (Table 5, entries 8 and 9) did not improve the catalytic efficiency of **ArM 2** at pH 7.0.

**Table 5:** Selected RCM reaction with hCAII-based artificial metathase ArM 2.

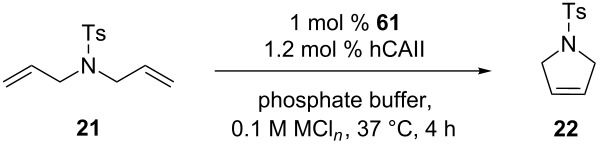				

entry^a^	hCAII^b^	MCl*_n_* (mol/L)	pH	TON

1 2 3 4 5 6 7 8 9	– WT – – WT – WT L198H L198H	MgCl_2_ (0.1) MgCl_2_ (0.1) – MgCl_2_ (0.5) MgCl_2_ (0.5) NaCl (0.154) NaCl (0.154) NaCl (0.154) –	6.0 6.0 7.0 5.0 5.0 7.0 7.0 7.0 7.0	48 ± 0.8 45 ± 2.0 23 ± 2.1 85 ± 1.0 78 ± 2.5 32 ± 2.0 21 ± 1.8 28 ± 0.6 22 ± 0.1

^a^Reaction conditions: [21]: 1 mM, [61]: 10 μM, [hCA II]: 12 μM, *V*_tot_: 200 μL (DMSO 10%), 37 °C. Reactions carried out in triplicate. ^b^WT = wild-type.

**Figure 3:**
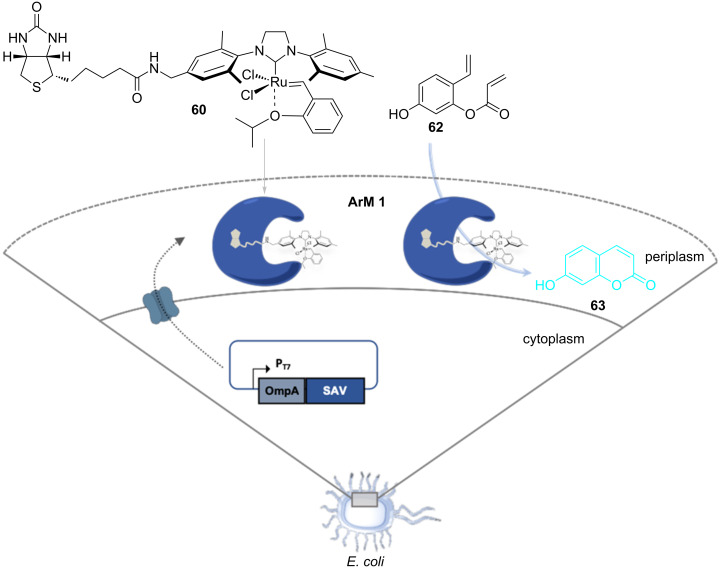
In vivo metathesis with an artificial metalloenzyme based on the biotin–streptavidin technology.

Jeschek et al. subsequently evolved **ArM 1** in vivo by directed evolution of an artificial metathase [68]. Tethering an OmpA leader sequence to the *N-*terminus of streptavidin (Sav) allowed the secretion and assembly of functional tetrameric Sav in the periplasm of *E. coli*. The passive diffusion of the biotinylated Hoveyda–Grubbs catalyst **60** through the outer membrane of *E. coli* containing Sav in its periplasm then affords the artificial metathase **ArM 1**. Upon addition of the umbelliferone precursor **62**, RCM reaction occurs in AcONa/AcOH buffer (pH 4.0) in the presence of 0.5 M MgCl_2_. The formed umbelliferone (**63**) can be detected by fluorescence (Figure 3).

The fifth generation Sav-mutant resulting from directed evolution (Sav_mut^5*^) displayed a cell-specific activity 5.4 ± 1.2 times higher than the wild-type enzyme. Table 6 summarizes the different RCM reactions tested using purified **ArM 1** in aqueous buffer at 37 °C [68–69].

**Table 6:** Selected RCM results obtained with artificial metathase **ArM 1** using purified Sav samples.

entry^a^	substrate	catalyst (%)	protein^b^	TON

1	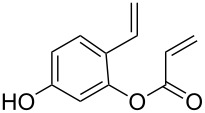 **62**	**60**	–	1.1
2	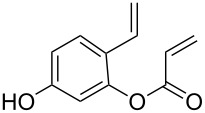 **62**	**60**	Sav	1.7
3	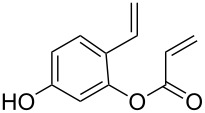 **62**	**60**	Sav_mut^5*^	4.4
4	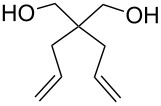 **64**	**60**	–	180 ± 4^c^
5	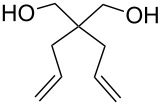 **64**	**60**	Sav	430 ± 3^c^
6	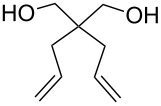 **64**	**60**	Sav_mut^5*^	650 ± 35^c^
7^d^	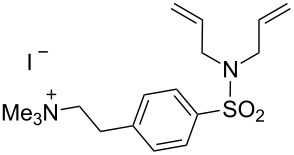 **65**	**60**	–	30 ± 1
8^d^	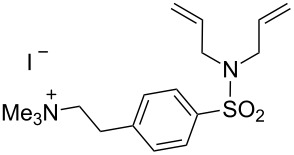 **65**	**60**	Sav	52 ± 2
9^d^	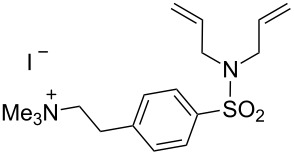 **65**	**60**	Sav_mut^5*^	90 ± 3

^a^Reaction conditions: 100 mM acetate buffer, 0.5 M MgCl_2_, pH 3.6, [catalyst] = 50 µM, 16 h at 37 °C and 200 rpm. ^b^Sav_mut^5*^ = Sav V47A/N49K/T114Q/A119G/K121R. ^c^TON determined by ^1^H NMR. ^d^[Substrate] = 20 mM; TON determined by UPLC–MS analysis.

**Scheme 14:**
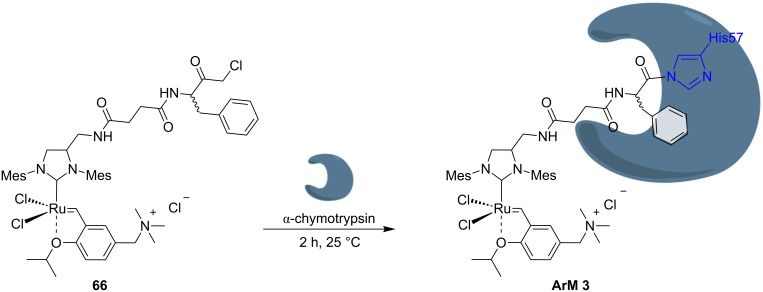
Artificial metathase based on covalent anchoring approach. α-Chymotrypsin interacts with catalyst **66** through supramolecular interactions followed by covalent nucleophilic attack to afford **ArM 3**.

Matsuo et al. used α-chymotrypsin as protein scaffold to assemble an artificial metathase by covalent anchoring [70]. α-Chymotrypsin is a serine protease that recognizes hydrophobic residues in one of its clefts. A modified HG-type catalyst (**66**) contains an L-phenyl chloromethyl ketone moiety that acts as inhibitor and is first recognized by supramolecular anchoring and then covalently attaches upon nucleophilic attack at the chloromethyl moiety by the imidazole of His57, to afford the artificial metathase **ArM 3** (Scheme 14).

Matsuo et al. tested the RCM of three different substrates with the protein-free catalyst **66** as well as **ArM 3** (Table 7). No RCM occurred with substrate **52** (<2 TON) with catalyst **66**, while the RCM of **67** reached 20 and 14 TON, respectively, with **ArM 3** and catalyst **66**. However, **ArM 3** decreased the RCM activity of **21** to 4 TON compared to 20 TON with catalyst **66**.

**Table 7:** RCM activities of catalyst **66** and **ArM 3** with substrates **67**, **52** and **21**.

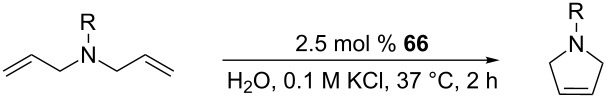			

entry	substrate	catalyst	TON

1	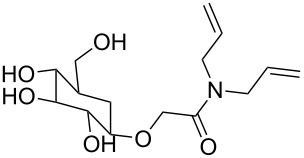 **67**	**ArM 3**	20
2	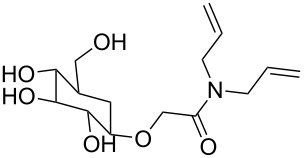 **67**	**66**	14
3	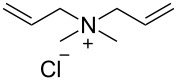 **52**	**ArM 3**	N.D.
4	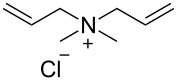 **52**	**66**	<2
5	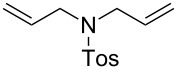 **21**	**ArM 3**	4
6	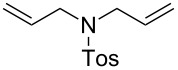 **21**	**66**	20

**Scheme 15:**
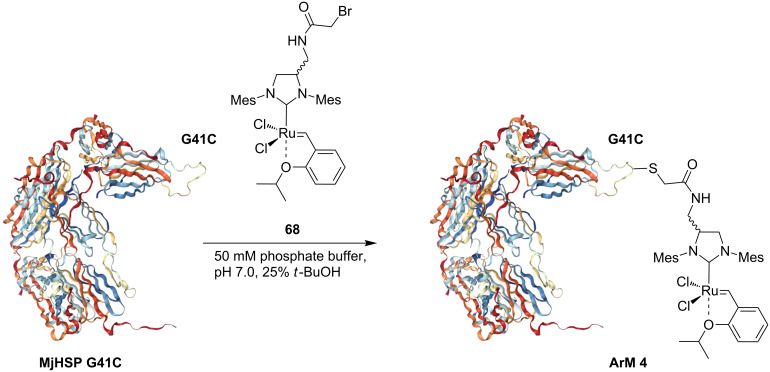
Assembling an artificial metathase (**ArM 4**) based on the small heat shock protein from *M. Jannaschii* (MjHSP). The protein structure is based on the atomic coordinates in PDB entry 1SHS.

In 2011, Hilvert and co-workers reported an ArM based on the covalent anchoring of a metathesis catalyst to a small heat shock protein from *M. Jannaschii* (MjHSP) [71]. The authors reported a HG-II-type catalyst modified on its NHC backbone with an α-bromoacetyl unit (**68**) that is reacted with the unique cysteine of the modified MjHSP variant (G41C) to afford **ArM 4** (Scheme 15).

The hybrid catalyst **ArM 4** was then tested for the aqueous RCM of substrate **21**. In a H_2_O/*t*-BuOH mixture, the catalytic efficiency of **ArM 4** markedly increases upon lowering the pH (Table 8, entry 6), although under the same conditions, the free catalyst **68** performs better (Table 8, entry 3).

**Table 8:** RCM of *N*,*N*-diallyltoluenesulfonamide (**21**) with **ArM 4**.

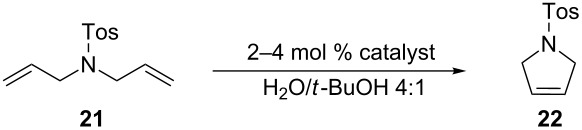				

entry	catalyst (mol %)	buffer	pH	TON

1 2 3 4 5 6	**68** (2) **68** (2) **68** (2) **ArM 4** (4) **ArM 4** (4) **ArM 4** (4)	50 mM phosphate 50 mM MES 10 mM HCl 50 mM phosphate 50 mM MES 10 mM HCl	7.0 3.9 2.0 7.0 3.9 2.0	2 ± 0.2 16 ± 0.4 33 ± 0.5 3 ± 0.1 12 ± 1.5 25 ± 2.1

**Scheme 16:**
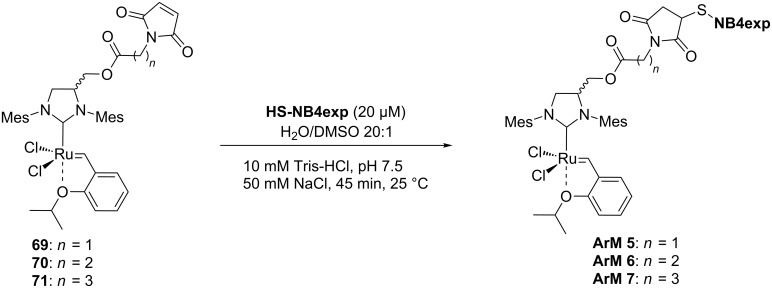
Artificial metathases based on cavity-size engineered β-barrel protein nitrobindin (**NB4exp**). The HG-type catalysts **69**, **70** and **71** are located inside nitrobindin to afford **ArM 5**, **ArM 6** and **ArM 7**.

Cavity-size engineered ArMs are the first example of biohybrid catalysts able to catalyze all three main olefin metathesis reactions (RCM, ROMP and CM) [72]. Schwaneberg and Okuda engineered the cavity size of the β-barrel protein nitrobindin (variant 4, NB4) to accommodate HG-type catalysts. The authors followed a similar approach developed earlier with a variant of the β-barrel protein FhuA [73–74]. To do so, the authors duplicated multiple β-barrel strands to enlarge the cavity of the protein. HG-type catalysts bearing a maleimide moiety with different spacer lengths (**69**–**71**) were covalently anchored to a cysteine of the expanded nitrobindin variant (**NB4exp**). The coupling reaction in aqueous buffer at pH 7.5 finally affords **ArM 5**, **ArM 6** and **ArM 7**, respectively (Scheme 16).

The obtained hybrid catalysts were tested for the RCM with substrates **21** and **64** (Table 9). Overall, **ArM 6** and **ArM 7** are comparable and perform best in both reactions with 35% conversion of substrate **21** and quantitative conversion of substrate **64**. The water-soluble catalyst **9** was compared to the hybrid catalysts, displaying a higher TON in the RCM of **21** (Table 9, entry 4). Interestingly, the activity of catalyst **9** is inhibited in the presence of **NB4exp** (Table 9, entries 5 and 10).

**Table 9:** Selected RCM results of *N*,*N*-diallyltoluenesulfonamide (**21**) and diol **64**.

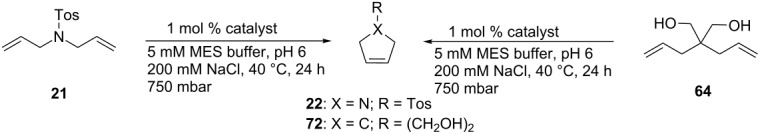			

entry	catalyst	substrate	conversion (%) = TON

1	**ArM 5**	**21**	16
2	**ArM 6**	**21**	35
3	**ArM 7**	**21**	35
4	**9**	**21**	41
5	**9** + **NB_4_exp**	**21**	0
6	**ArM 5**	**64**	45
7	**ArM 6**	**64**	100
8	**ArM 7**	**64**	100
9	**9**	**64**	100
10	**9** + **NB_4_exp**	**64**	0

In the ROMP of the norbornene derivative **13**, **ArM 6** and **ArM 7** performed best, outperforming catalyst **9**. A near ten-fold increase is observed for **ArM 6** (Table 10, entry 2).

**Table 10:** ROMP of 7-oxonorbornene derivative **13** with β-barrel engineered artificial metalloenzymes.^a^

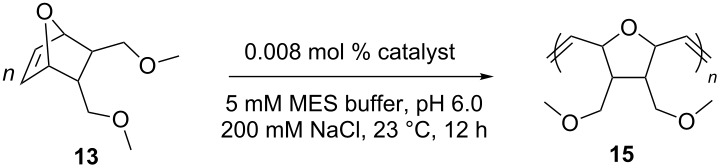				

entry	catalyst	conversion^b^ (%)	TON	PDI^c^

1	**ArM 5**	25	3000	1.29
2	**ArM 6**	81	10000	1.21
3	**ArM 7**	75	9300	1.29
4	**9**	16	1700	N.D.

^a^[13] = 0.2 M. ^b^Determined by ^1^H NMR spectroscopy. ^c^PDI = polydispersity index.

In the cross metathesis of terminal olefins **73**, **74**, and **75**, with the commercial catalyst **9** conversions of 79%, 98% and 94%, respectively, were achieved. As in the RCM, the combination with **NB4exp** did not give any conversion (Table 11, entry 5). All three ArMs converted the three substrates with good yields of products **76**, **77** and **78**. **ArM 6** performed the best, affording quantitative conversion for all three substrates (Table 11, entries 2, 7 and 12).

**Table 11:** Selected CM results with cavity-size engineered ArMs.

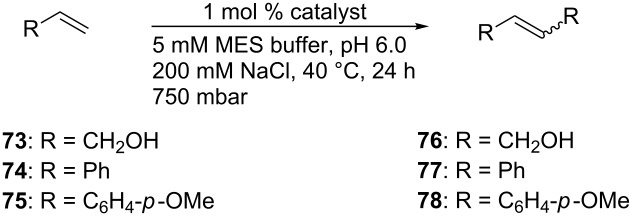				

entry^a^	catalyst	substrate	conversion^b^ (%)	TON

1	**ArM 5**	**73**	>99^c^	100
2	**ArM 6**	**73**	>99^c^	100
3	**ArM 7**	**73**	69^c^	69
4	**9**	**73**	79^c^	79
5	**9** + **NB_4_exp**	**73**, **74**, **75**	0	0
6	**ArM 5**	**74**	45^c^	45
7	**ArM 6**	**74**	>99^c^	100
8	**ArM 7**	**74**	>99^c^	100
9	**9**	**74**	98^d^	98
10	**ArM 5**	**75**	40^d^	40
11	**ArM 6**	**75**	>99^d^	100
12	**ArM 7**	**75**	>99^d^	100
13	**9**	**75**	94^d^	94

^a^[Substrate] = 0.05 M. ^b^Conversions determined by ^1^H NMR. ^c^*E*/*Z* = 20:1. ^d^*E*/*Z* = 99:1.

**Scheme 17:**
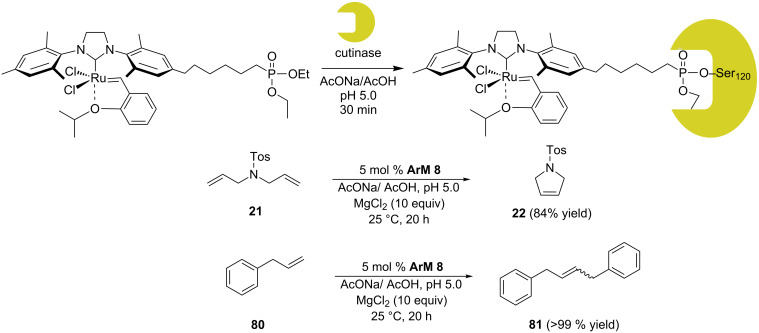
Artificial metathase based on cutinase (**ArM 8**) and resulting metathesis activities.

Gebbink and co-workers anchored the HG-type catalyst **79** to cutinase, a serine hydrolase [75]. The phosphonate ester moiety acts as a suicide inhibitor forming an irreversible covalent bond to a serine residue present in the active site of the enzyme. Assembly of **ArM 8** occurs at pH 5 (Scheme 17). The activity of the artificial metalloenzyme was tested with the benchmark RCM substrate **21**, yielding 84% of product **22** in acetate buffer at pH 5 (TON = 16.8). The same conditions were applied to the self-metathesis of substrate **80**, affording a quantitative conversion (Scheme 17).

### Olefin metathesis: applications in chemical biology

Synthetic compounds are increasingly being used as chemical tools to scrutinize and modulate biological systems [76]. Olefin metathesis is a prime example of bioorthogonal reactions and the ruthenium catalysts display good stability and chemoselectivity. The first applications of olefin metathesis in chemical biology were reported with “ill-defined” catalysts such as RuCl_3_·H_2_O to synthesize insect pheromones by olefin metathesis [77–78]. The development of well-defined ruthenium-based catalysts increased the number of olefin metathesis applications in chemical biology thanks to their tolerance against various functional groups such as amides, alcohols and carboxylic acids. However, one major hurdle for olefin metathesis in chemical biology remains the necessity to perform catalysis under mild conditions in buffered aqueous media.

The aqueous ROMP introduced by Grubbs and co-workers led to several biological applications [79–80]. Kiessling and co-workers were the first to use ROMP for the synthesis of biologically active polymers and for the synthesis of multivalent antigens to probe signaling pathways in vivo [81–82].

**Scheme 18:**
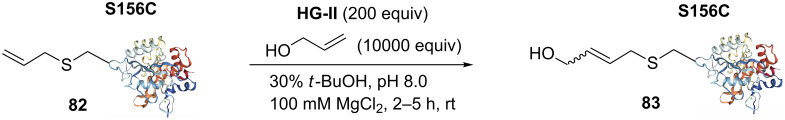
Site-specific modification of proteins via aqueous cross-metathesis. The protein structure is based on the atomic coordinates in PDB entry 1NDQ.

In 2008, Davis and co-workers performed site-selective protein modification through aqueous CM [83], thus expanding the catalytic repertoire of protein modification with transition-metal catalysts [84–87]. A variant of subtilisin from *Bacillus lentus* containing a single cysteine (SBL-S156C) was modified by direct allylation to install an allyl-sulfide on the surface of the protein. Cross metathesis of the modified protein **82** with allyl alcohol gave the CM product with over 90% conversion (Scheme 18).

To achieve this challenging reaction, 200 equivalents (equiv) of **HG-II** catalyst were employed in a reaction mixture containing 0.01 mM **82**. Remakably, no conversion was observed in the absence of MgCl_2_, which prevents the non-productive binding of the amino acid side chains to ruthenium. The authors suggested that the positive effect of allyl sulfides may be due to the coordination of the sulfur atom to the ruthenium center, favoring the formation of the metallacyclobutane intermediate. The modest activities of butenyl and pentenyl sulfides were rationalized by the formation of five and six-membered ring chelates. The aqueous CM with allyl sulfides was also exploited by Hunter et al. for the generation of a metathesis-based dynamic combinatorial library [88].

**Scheme 19:**
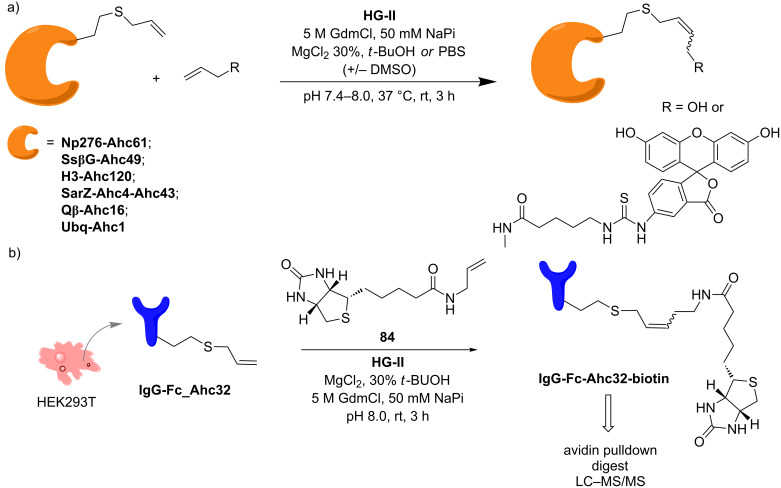
a) Allyl homocysteine (**Ahc**)-modified proteins as CM substrates. b) Incorporation of Ahc in the Fc portion of IgG in human cells (HEK 293T) and CM reaction with **84**.

The work carried out by Davis and co-workers led to the metabolic incorporation of unnatural amino acids (uAAs) bearing a terminal alkene as CM substrates for protein modification [89]. The authors investigated the possibility to incorporate methionine (Met) analogues in a Met-auxotrophic strain of *E. coli* (B834DE3). Allyl-homocysteine (**Ahc**) resulted in the only uAA successfully incorporated into 6 different proteins, namely Histone H3 (**H3-Ahc120**), Np276 (**Np276-Ahc61**), SsβG (**SsβG-Ahc49**), SarZ (**SarZ-Ahc4-Ahc43**), Qβ (**Qβ-Ahc16**), and Ubq (**Ubq-Ahc1**). The modified proteins were tested for cross metathesis with allyl alcohol or with a fluorescein derivative (Scheme 19a).

To further advance the chemical tagging through cross metathesis, genetic incorporation of **Ahc** was performed in human cells (HEK 293T) for the modification of the Fc region of IgG (IgG-Fc-Ahc32, Scheme 19b). An olefin-bearing biotin **84** was selected as olefinic partner for the CM reaction with the modified antibody, yielding **IgG-Fc-Ahc32-biotin** (Scheme 19b). The conjugated protein can be selectively pulled-down with avidin beads and analyzed by tandem MS after tryptic digestion. This strategy suggests that CM reactions can be integrated in the toolbox of chemical proteomics.

Recently, following a similar strategy, Lu et al. reported on-DNA RCM and CM, an application potentially useful to generate DNA-encoded libraries for hit identification and target validation [90]. Substrates appended to oligonucleotides undergo Ru-promoted RCM and CM when the **G-III** catalyst is used under heterogeneous conditions (water/*tert*-butanol 3:2) with a large excess of Mg^2+^. Also in this case, the role of Mg^2+^ is to protect the oligonucleotide from Ru-induced decomposition by binding to the phosphate backbone. Table 12 summarizes the activities of 7 different DNA-tethered substrates for RCM. Good conversions were achieved in water mixtures (40% *t*-BuOH) at room temperature after 1 hour of reaction. However, these reactions are not catalytic as they require 150 equivalents of the **G-III** catalyst.

**Table 12:** Scope of RCM reactions using DNA-tethered substrates.^a^

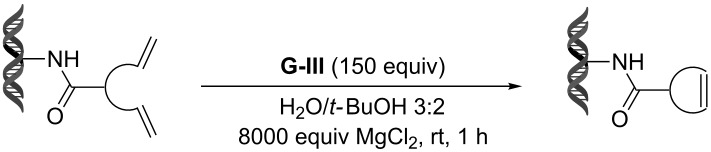			

entry	substrate	product	conversion (%)

1	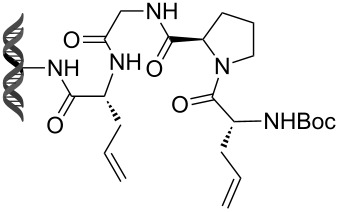 **85**	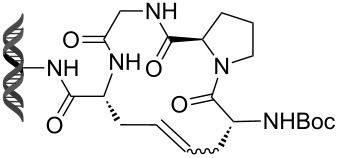 **86**	50
2	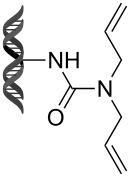 **87**	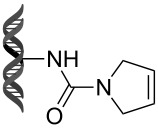 **88**	85
3	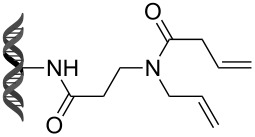 **89**	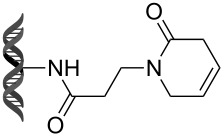 **90**	65
4	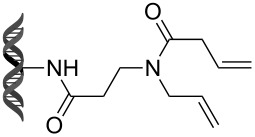 **91**	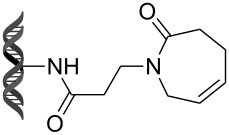 **92**	65
5	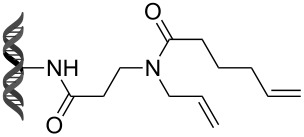 **93**	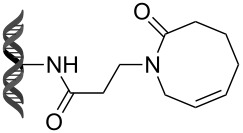 **94**	50
6	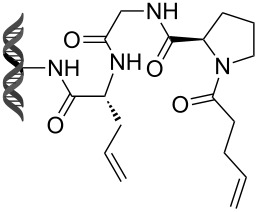 **95**	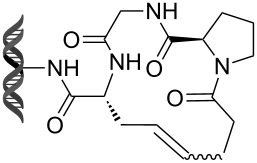 **96**	55
7	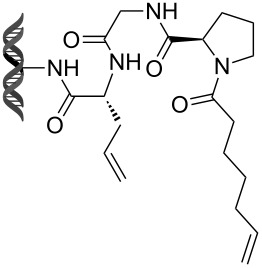 **97**	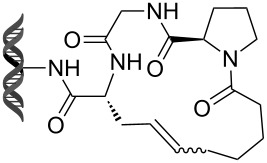 **98**	50

^a^150 equiv **G-III** catalyst, [substrate] = 0.09 mM.

**Scheme 20:**
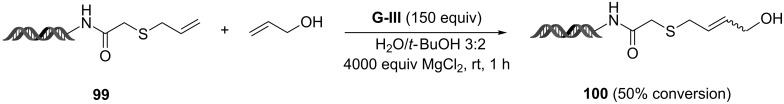
On-DNA cross-metathesis reaction of allyl sulfide **99**.

The same conditions were tested for the cross metathesis of the allyl-sulfide **99** with allyl alcohol, yielding 50% of product **100** in aqueous mixture (40% *t*-BuOH) in the presence of 4000 equiv of Mg^2+^ (Scheme 20).

**Scheme 21:**
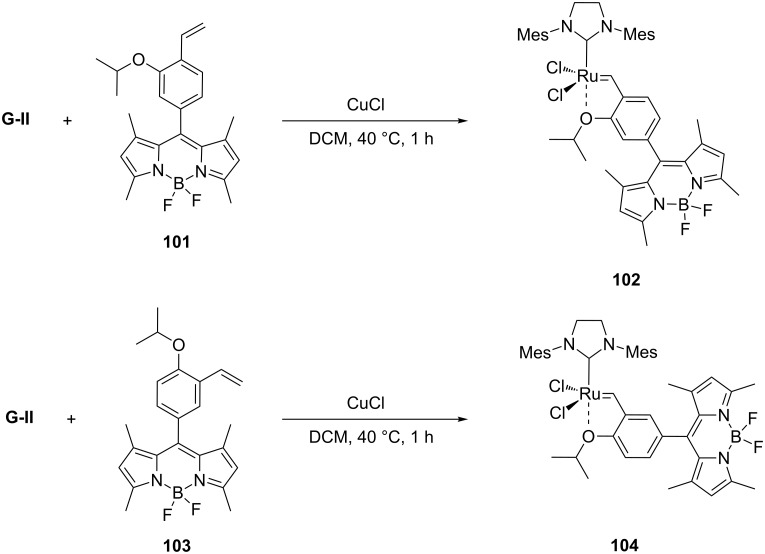
Preparation of BODIPY-containing profluorescent probes **102** and **104**.

In another recent study, Touissant et al. described the synthesis of two metathesis-based fluorescent probes suitable for the detection of ethylene in live cells [91]. BODIPY fluorophores bearing the isopropyloxybenzylidene moiety (**101** and **103**) reacted with the **G-II** catalyst to form the **HG-II** derivatives **102** and **104**, respectively (Scheme 21).

**Scheme 22:**
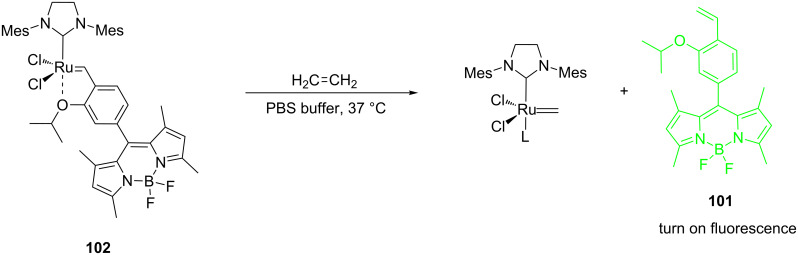
Metathesis-based ethylene detection in live cells.

The resulting compounds are Ru-based profluorescent probes that become fluorescent in the presence of ethylene, thus leading to the release of **101** from the Ru-catalyst (Scheme 22).

Live cell experiments with *Chlamydomonas reinhardtii* suggest that 20 µM of probe **102** in PBS buffer are sufficient to turn fluorescence on in cells flushed with exogenous ethylene or ethylene gas derived from ripe fruit (e.g., banana or mango). Control experiments reveal however a steady increase in fluorescence in the absence of ethylene, suggesting that further optimization of the probes is required. As ethylene plays an important role as a plant hormone, metathesis-based probes might have interesting applications in plant biology.

**Scheme 23:**
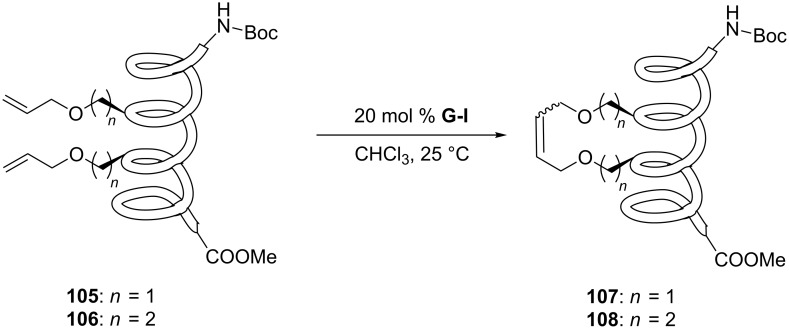
First example of stapled peptides via olefin metathesis.

Olefin metathesis is also used to cross-link peptide fragments. This technology is known as peptide stapling [92]. Blackwell et al. engineered the first stapled peptide in 1998 by introducing two non-natural amino acids bearing a terminal alkene in a peptide sequence (e.g., **105**, **106**) [93]. The cross-linking of the two amino acids by metathesis results in a more rigid and stabilized alpha helix (products **107** and **108**, Scheme 23).

Although the reaction cannot be classified as aqueous metathesis (the reaction is carried out in CHCl_3_ and the peptide remains attached to the solid phase), this technology has been exploited to disrupt protein–protein interactions (PPIs) in cancer cells [94–96]. Aileron Therapeutics recently launched a stapled peptide platform aiming at developing molecules like ALRN-6924, a stapled peptide that interacts with p53 inhibitors MDMX and MDM4. The drug candidate is currently being evaluated in clinical trials for different types of cancer [97].

## Conclusion

Over the past 20 years, the number of applications of olefin metathesis in water has dramatically increased. The field of metathesis is continuously growing and scientists seek new opportunities to exploit this powerful C–C double-bond-forming reaction in different fields of research. Several biological applications have emerged over the past 10 years as a result of the extensive efforts to establish biocompatible protocols. While aqueous metathesis offers the advantage of performing catalysis in a more sustainable medium, it still remains challenging to achieve due to the detrimental effect of water. Despite this limitation, olefin metathesis widely contributes to polymer chemistry, drug discovery and biocatalysis. Several technologies relying on aqueous metathesis have been developed (e.g., protein modification, on-DNA metathesis, directed evolution of artificial metalloenzymes, etc.) and are paving the way to future interesting applications.
